# A growth selection system for sucrose synthases (SuSy): design and test

**DOI:** 10.1186/s13568-024-01727-y

**Published:** 2024-06-12

**Authors:** Gonzalo N. Bidart, Se Hyeuk, Tobias Benedikt Alter, Lei Yang, Ditte Hededam Welner

**Affiliations:** 1 The Novo Nordisk Center for Biosustainability, Technical University of Denmark, Kemitorvet 220, Kgs. Lyngby, DK-2800 Denmark; 2 Present Address: RWTH Aachen University, Templergraben 55, 52062 Aachen, Germany

**Keywords:** Growth-couple selection system, Enzyme engineering, Sucrose synthase, *Escherichia coli*

## Abstract

High throughput screening (HTS) methods of enzyme variants are essential for the development of robust biocatalysts suited for low impact, industrial scale, biobased synthesis of a myriad of compounds. However, for the majority of enzyme classes, current screening methods have limited throughput, or need expensive substrates in combination with sophisticated setups. Here, we present a straightforward, high throughput selection system that couples sucrose synthase activity to growth. Enabling high throughput screening of this enzyme class holds the potential to facilitate the creation of robust variants, which in turn can significantly impact the future of cost effective industrial glycosylation.

**Supplementary Information:**

The online version contains supplementary material available at 10.1186/s13568-024-01727-y.

## Introduction

Glycosides are widely distributed in nature and can be found in nearly every living organism (Lis and Sharon 1993). In plants, glycosides are part of the secondary metabolism and play a role in different processes like accumulation, storage, and transport of hydrophobic substances, as precursors for flower fragrances(Watanabe et al. 1993) and UV radiation protection (Hofmann 2000). Glycosylation of lipophilic low molecular weight compounds is an efficient tool to reduce chemical toxicity, enhance water solubility and improve stability (Fu et al. 2019), and hence positively affect shelf life and bioavailability of various chemicals and pharmaceuticals(Bowles et al. 2006; Seeberger et al. 2022). Additionally, glycosides themselves have industrial applications, *e.g.* in detergents and cosmetics (de Roode et al. 2003), as antimicrobials (Sahari and Asgari 2013), as dyes (Hsu et al. 2018), and aroma precursors (Schwab et al. 2015).

Glycosides can be produced chemically or enzymatically. In general, enzymatic glycosylation offers several advantages over chemical synthesis, including high efficiency, high degree of regio, stereo and chemoselectivity, and mild reaction conditions (Lairson et al. 2008; Putkaradze et al. 2021). In nature, the main enzyme class responsible for glycosylation of small molecules are the uridine diphosphate (UDP) dependent glycosyltransferases (UGTs), members of family 1 in the Carbohydrate Active Enzymes database (Lombard et al. 2014). They are Leloir glycosyltransferases (Lairson et al. 2008), transferring a sugar from an activated nucleoside diphosphate (NDP)-sugar donor to a small lipophilic acceptor. The central challenge of biocatalytic industrial glycosylation lies in securing an abundant supply of the activated sugar donor, such as uridine 5’-diphosphate glucose (UDP-Glc), which are scarce, expensive, but essential for Leloir GT catalyzed glycosylation. Sucrose synthases (SuSy) can play a central role in the provision of UDP-Glc (Diricks et al. 2016; Schmölzer et al. 2016). These enzymes, members of GT family 4, catalyzing the reversible synthesis of sucrose from fructose and NDP-glucose, allow for the recycling of the NDP-sugar donor by pushing the thermodynamic equilibrium, using abundant and cheap sucrose. SuSys have already been applied at lab scale together with different UGTs in one pot reaction systems to produce a variety of glycosides, including steviol glycosides (Chen et al. 2018), glycyrrhetinic acid glucoside (Ali et al. 2021), polydatin(Chen et al. 2021), nothofagin (Putkaradze et al. 2023), indican (Bidart et al. 2024), and orientin and vitexin (Gu et al. 2021).

In order to achieve the high titers required for glycosylation reactions at scale, organic solvents are usually applied to help dissolve the hydrophobic substrates (Pei et al. 2019; Tao et al. 2023). In this context, SuSy stability seems to be challenged, limiting further industrial implementation (Diricks 2017; Liu et al. 2022; Orrego et al. 2017; Trobo-Maseda et al. 2020; Zhao et al. 2023). In recent years, multiple studies have focused on improvement of SuSy stability using different approaches such as rational(Diricks 2017) and semi rational (Zhao et al. 2023) enzyme engineering, immobilization (Liu et al. 2022; Orrego et al. 2017; Trobo-Maseda et al. 2020), and enzyme mining(Chen et al. 2023), to find a robust variant suitable for industrial application. So far, enzyme engineering has successfully led to variants with increased kinetic stability, providing longer half lives, however this did not translate in higher thermo or chemostability (Zhao et al. 2023). Enzyme immobilization was also successful at increasing the stability compared to free enzyme, although with a concomitant activity loss (Liu et al. 2022; Orrego et al. 2017; Trobo-Maseda et al. 2020). Lastly, with the rapid increase in DNA sequence data, enzyme mining has emerged as an attractive approach to find robust variants. This is the case of the SuSy identified in the green algae *Micractinium conductrix* (*Mc*SuSy). *Mc*SuSy showed an attractive DMSO tolerance, however its kinetic stability is strongly reduced at temperatures above 50 °C (Chen et al. 2023).

*In vivo* growth couple selection systems are a versatile methodology for directed evolution of enzymes (Luo et al. 2019; Neuenschwander et al. 2007; Wu et al. 2022). By coupling the activity of a targeted enzyme with growth of a host organism, *in vivo* selection allows simple and robust identification of active variants within a large library. Although several pathways for microbial sucrose utilization are known, and despite the industrial relevance of sucrose utilizing enzymes (as SuSy), *in vivo* growth coupled selection systems for engineering of these industrial biocatalyst have never been demonstrated. In this work we report a generally applicable growth coupled selection system for the engineering of robust SuSy variants.

## Materials and methods

### Bacterial strains, medium and growth conditions

The strains used in this study are listed in Table 1. *E. coli* MGcscBKA strain and derivatives were used in this study. Genome engineering of *E. coli* MGcscBKA was performed by CRISPR/MAD7 method as described previously(Phaneuf et al. 2021). *E. coli* DH5α™ subcloning efficiency™ (ThermoFisher Scientific, Germany), was used as host in cloning experiments. All strains were mantained in Luria–Bertani (LB) medium at 37 °C under agitation, and transformants were selected with Chloramphenicol (25 µg mL^−1^). Growth curves were done on M9 minimal medium following the protocol described below.

### Plasmids

The plasmids used in this study are listed in Table 1. DNA fragments containing the Sucrose Synthases genes were obtained by PCR using pET28a + plasmids encoding the targeted enzymes (purchased from Genscript, United States) as templates and the corresponding oligonucleotide pairs. The PCR products were purified using a NucleoSpin Gel and PCR kit (Macherey-Nagel, Germany) and cloned into the backbone of pMTP7 (obtained by PCR using primers pMPT7-Fw and pMPT7-Rv) by USER cloning (New England Biolabs, US). Chemically competents *E. coli* DH5α™ were transformed by heat shock with 3 µl of the USER reactions and after 1 h recovery cells were plated on LB with Chloramphenicol. The resulting plasmids were purified using a NucleoSpin Plasmid kit (Macherey-Nagel, Germany), and its sequence verified (Eurofin Genomics). *E. coli* SDT 365 electrocompetents were transformed with each of these plasmids using a single electric pulse in a BioRad MicroPulser as indicated by the manufacturer (program Ec2; 10 mF, 600 Ω, 12,5 kV/cm).

**Table 1:** Strains and plasmids used in this study

Strain	Relevant genotype or properties	Source
*E. coli* DH5α™	F- Φ80*lacZ*ΔM15 Δ(*lacZYA-argF*) U169 *recA1 endA1 hsdR17*(rk-, mk+) *phoA supE*44 *thi-1 gyrA*96 *relA1* λ-	ThermoFisher Scientific
EESB 1	*E. coli* DH5α™ pMTP7-GmSuSy (Cm^R^)	This work
EESB 2	*E. coli* DH5α™ pMTP7-AcSuSy (Cm^R^)	This work
EESB 3	*E. coli* DH5α™ pMTP7-AcSuSy L637M-T640V (Cm^R^)	This work
MGcscBKA	K-12 MG1655 torS-cscBKA	(Mohamed et al. 2019)
SDT 365	MGcscBKA Δ*cscA* Δ*otsA*	This work
EESB 4	SDT 365 pMTP7-GmSuSy (Cm^R^)	This work
EESB 5	SDT 365 pMTP7-AcSuSy (Cm^R^)	This work
EESB 6	SDT 365 pMTP7-AcSuSy L637M-T640V (Cm^R^)	This work

Plasmid	**Description**	**Source**
pSD85	P_j23119_-gRNA_*cscA*-*otsA*, Cm^R^, pBR322	This study
pGE3	*araC*, P_ara_-*gam*-*bet*-*exo*, tet^R^, P_lac_I-*tetO*, P_tet_-gRNA_pBR322, P_j23105_-MAD7, Amp^R^, SC101(ts)	(Phaneuf et al. 2021)
pGNB1	pMTP7- carrying GmSuSy (Cm^R^)	This work
pGNB2	pMTP7- carrying AcSuSy (Cm^R^)	This work
pGNB3	pMTP7- carrying AcSuSy L637M-T640V (Cm^R^)	This work

Cm^R^, Chloramphenicol resistance

### DNA manipulation, oligonucleotides and sequencing

PCR primers (Supplemental Table 1) were synthesized by IDT (USA). SuSy-SeqFw and SuSy-SeqRv backbone primers were used for checking plasmid assembly. All PCR reactions were performed with the Phusion U Hot Start polymerase (ThermoFisher Scientific, Germany). DNA sequencing was carried out by Eurofins Scientific (Luxembourg). Sequence analyses were carried out with SnapGene Viewer (Dotmatics) and sequence similarities were analyzed with BLAST3.

### Metabolic model based analysis

The growth coupling design for SuSy was confirmed and analyzed using iML1515(Monk et al. 2017), a comprehensively curated genome scale metabolic model for *E. coli* K-12 MG1655. ADP, UDP dependent variants of SuSy, and the proton symporting sucrose permease were added to iML1515 to enable the uptake and metabolization of sucrose. To disable trehalose-6-phosphate synthase activity (UDP recycling), thus resembling the genetic background of the strain SDT365, *otsA* (b1896) was knocked out in the model. Note that the gene *cscA* and its associated metabolic reactions are not present in iML1515. Flux balance analyses were conducted assuming a maximum sucrose and oxygen uptake rate of 7.5 mmol gDW^−1^ h^−1^ and 20 mmol gDW^−1^ h^−1^, respectively.

Model handling and all simulations were performed using the COBRApy toolbox (Ebrahim et al. 2013) (version 0.26.2) and the Gurobi optimization suite (GurobiOptimization 2023) (version 9.0.2). A Jupyter notebook including all relevant *in silico* analyses is provided in the Supplementary information.

### Genome engineering of parental strain MGcscBKA

Simultaneous knockout of *cscA* and *otsA* from sucrose utilizing MG1655 derived strain, EMP4, was achieved by CRISPR/MAD7 method as described in a previous study(Phaneuf et al. 2021) with a slight modification. gRNA plasmid containing both *cscA* and *otsA* targeting gRNA sequence (pSD85) was constructed by USER cloning(Cavaleiro et al. 2015) for multiplex gene editing. gRNA plasmid backbone was amplified by a pair of primers, SD_PR356 and SD_PR357 (Supplemental Table 1), to achieve the array 1 structure applied from Lin et al.(Lin et al. 2021). We followed the same procedure for genome engineering as the previous study, except that 100 pmol of two MAGE oligos (SD_PR361 and SD_PR364) for knocking out the *cscA* and *otsA* genes were mixed before being added. The knockout of genes was validated through colony PCR using flanking primers for each gene.

### Culture of SDT365 and derivatives on minimal medium

Precultures of SDT 365 strains carrying or not pMTP plasmids were grown in tubes overnight at 37 °C under agitation (200 rpm) on LB Medium. Overnight cultures were collected by centrifugation (1 min at 17,000 g), washed twice with M9 minimal medium, and used to inoculate the main culture on 96 well microplates at 0,1 OD_600nm_ on M9 supplemented with 20mM sucrose, and with or without supplemented 3mM UDP (Biosynth, Berkshire, UK) or ADP (Sigma-Aldrich, USA). M9 contained the following sterile solutions, 1x M9 Salts Solution, 2 mM MgSO4, 100 µM CaCl2, ammonium iron (III) citrate 0,0002% (w/v), 1x trace elements, and 1x Wolfe’s vitamin solution. 10x stock M9 Salts Solution contained, per litre 68 g Na2HPO4 anhydrous, 30 g KH2PO4, 5 g NaCl, and 10 g NH4Cl dissolved in MilliQ filtered water. 400x stock Trace element solution contains, per litre 50 mg ZnCl2, 30 mg MnCl2 4H2O, 300 mg BO3H3, 200 mg CoCl2, 10 mg CuCl2 2H2O, 20 mg NiCl2 6H2O and 30 mg Na2MoO4 2H2O. Finally, 500x stock Wolfe’s vitamin solution contained, per litre: 10 mg Pyridoxine HCl, 5 mg Thiamine HCl, 5 mg Riboflavin, 5 mg Nicotinic acid, 5 mg Ca-D-(+)pantothenate, 5 mg p-Aminobenzoic acid, 5 mg Thiotic acid (Dithiolane Pentanoic acid), 2 mg Biotin, 2 mg Folic acid, and 0.1 mg Vitamin B12. The microplate cultures were performed as previously descrived(Kim et al. 2020) with a few adaptations. Each well consisted of 200 µl culture cover with 50 µl mineral oil (Sigma-Aldrich, USA) to avoid evaporation. Microplate were incubated at 37 °C with alternating linear and orbital agitation in an Epoch2 microplate spectrophotometer (BioTek, Agilent, USA), and bacterial growth was monitored every 10 min for 72 h measuring OD_600nm_. At least three independent biological replicates for each growth curve were obtained. Results were expressed as means ± standard deviations.

### Figures and data analysis

**Fig. 1:**
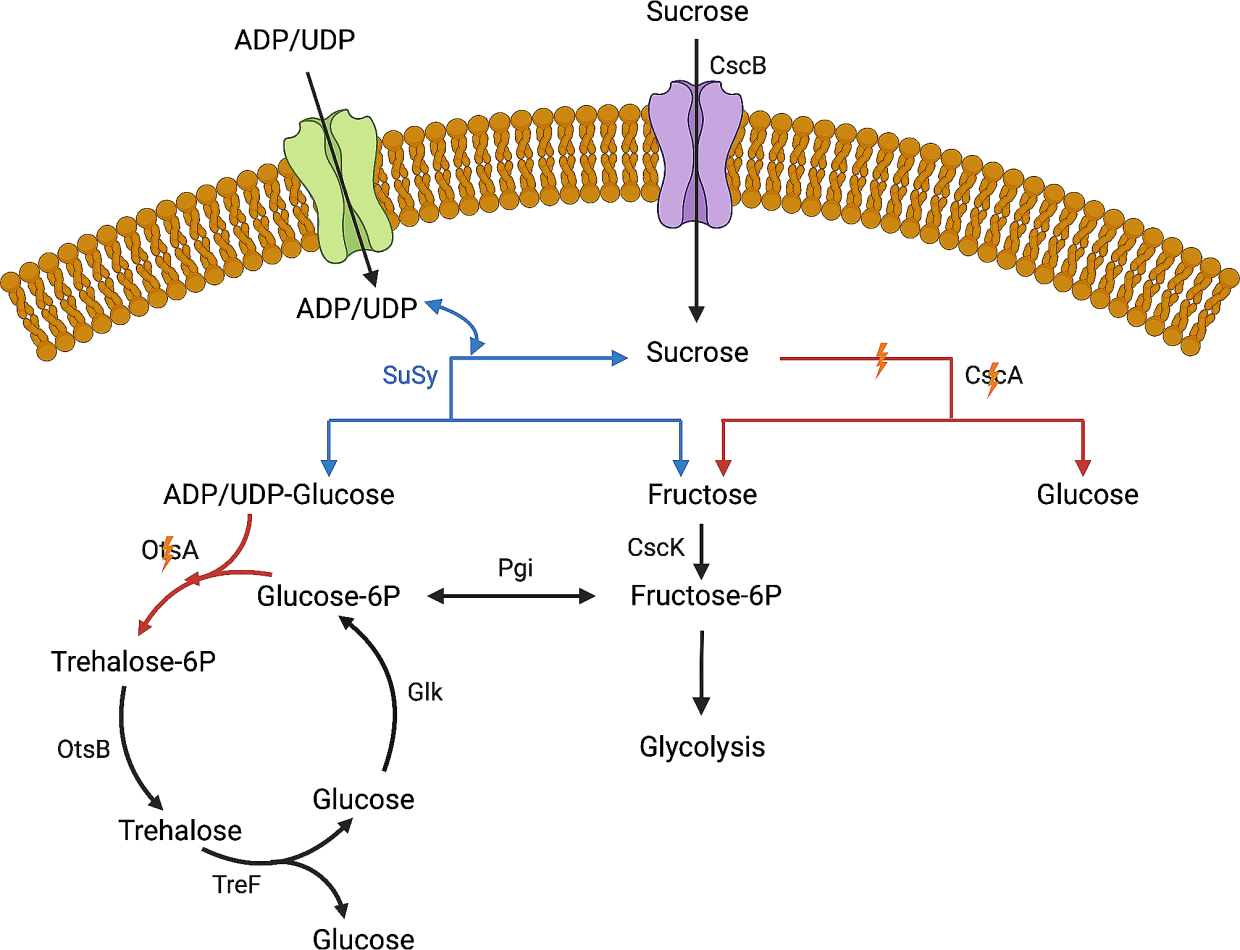
Illustration of growth coupled sucrose synthase selection system design in *E. coli* SDT 365.  Native reactions & enzymes are colored in black and heterologous in blue. Red lines indicate an inactive pathway after gene deletion (yellow bolt). CscB, sucrose permease; CscA, sucrose hydrolase; CscK, fructose kinase; Glk, glucokinase; Pgi, glucose-6P isomerase; SuSy, sucrose synthase; OtsA, trehalose-6P synthase; OtsB, trehalose-6P phosphatase; TreF, trehalase

**Fig. 2:**
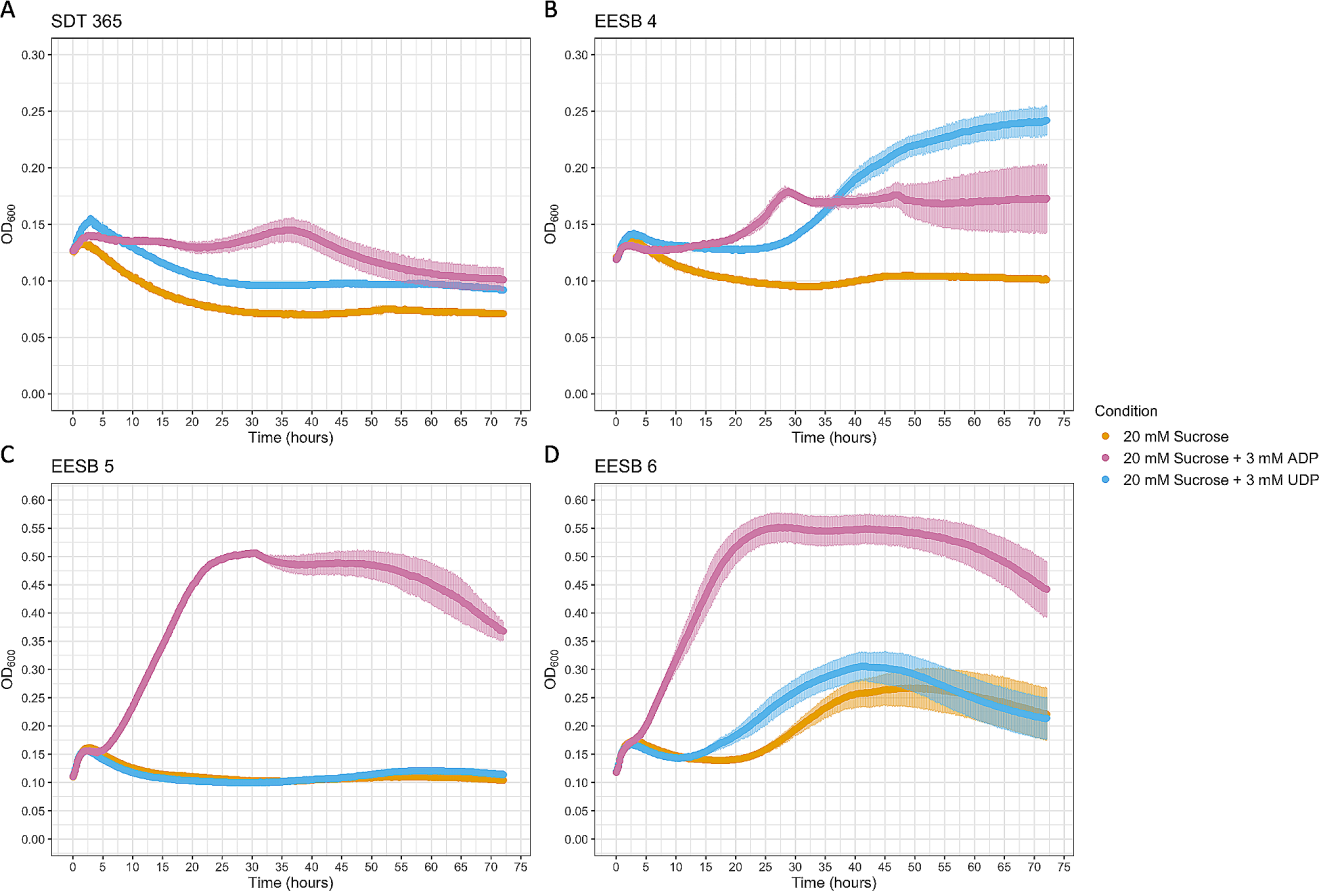
Growth curves of *E. coli* SDT 365 (without plasmid) (A), EESB 4 (B), EESB 5 (C), and EESB 6 (D) on M9 minimal medium supplemented with sucrose, and with or without (control) supplemented UDP/ADP. Data presented are average values based on at least three replicates. Error bars indicate standard deviations

Figure 1 and supplemental Fig. 1 was created with BioRender (https://www.BioRender.com). Data analysis and Fig. 2 were prepared in R (https://www.R-project.org/) using RStudio (https://www.RStudio.com).

## Results

### Design of a growth coupled selection system for SuSy variants

SuSy catalyzes the cleavage of sucrose into fructose and ADP/UDP-glucose in the presence of either NDP. To design a growth coupled selection system based on SuSy activity, we leveraged its byproduct fructose as the sole carbon source for microbial growth. Moreover, we took advantage of the previously optimized *E. coli* MGcscBKA strain for improved sucrose utilization (Mohamed et al. 2019), which contains genes for a sucrose permease (*cscB*) to allow the sucrose uptake, an invertase (*cscA*) to allow sucrose hydrolysis, and the fructose kinase (*cscK*) which allows fructose to directly enter glycolysis via fructose-6P (Fig. 1). We then knocked out *cscA* to avoid the utilization of sucrose as carbon source (Fig. 1). Therefore, when sucrose is supplied as the only carbon source in a minimal medium, only cells harboring an active sucrose utilizing enzyme can survive and grow on either glucose, fructose, or both, resulting from the sucrose utilizing reaction.

The design and growth rescue by SuSy activity was computationally validated using a genome scale metabolic model. The model confirms that SuSy restores growth when sucrose is provided as the sole carbon source, even without considering feeding ADP or UDP. If an extracellular ADP or UDP feed is added to the model and made solely available to SuSy, growth rates increase by 34% and 30%, respectively. These growth advantages demonstrate the benefit to the cell of using ADP/UDP recovered from ADP/UDP-glucose in metabolic processes other than SuSy activity. Furthermore, the model predicts a slightly higher growth rate (+ 6%) for an ADP specific SuSy in contrast to an UDP specific variant, since recycling of ADP from ADP-glucose by glycogen synthase (*glgA*) inflicts less metabolic burden on the cell than UDP recycling by the glucose-6-phosphate dependent trehalose-6-posphate synthase (*otsA*).

To prevent the synthesis of trehalose, a storage disaccharide that would otherwise act as a carbon sink in our selection scheme, the *otsA* gene was knocked out leading to strain SDT 365.

### Growth rescue by SuSy activity

We then proceeded to experimental validation. Growth curves of strain SDT 365 in M9 medium supplemented with sucrose confirmed that this strain is not able to grow on sucrose without harboring a complementary sucrose utilizing enzyme (Fig. 2A). Furthermore, the strain was neither able to grow on M9 medium supplemented with both sucrose and UDP, or sucrose and ADP (Fig. 2A). To test growth rescue by SuSy activity, SDT 365 was transformed with three vectors, each carrying a different SuSy variant under a constitutive strong promoter. These were the plant SuSy from *Glycine max* (*Gm*SuSy), the most frequently used SuSy so far, which has the strongest affinity towards UDP (*K*_M_ = 5 µM (Diricks et al. 2016), the bacterial SuSy from *Acidithiobacillus caldus* (*Ac*SuSy) which is more thermostable than plant SuSys, and has higher affinity towards ADP (*K*_M_ = 0.3 mM) compared with UDP (*K*_M_ = 7.8 mM) (Diricks et al. 2016); and a previously reported engineered variant of *Ac*SuSy (L637M-T640V) with increased affinity towards UDP (*K*_M_ = 0,13 mM) (Diricks et al. 2016), giving rise to the strains EESB 4, EESB 5 and EESB 6. Cultures of EESB 4 and EESB 5 showed no noticeable growth on a medium with only sucrose (Fig. 2B–C). Both strains partially restored growth when further supplemented with UDP or ADP (Fig. 2B–C), respectively, in accordance with their individual substrate preferences. In line with reported substrate affinities, strain EESB 6, which carry the engineered variant of *Ac*SuSy with high affinity towards both ADP and UDP, exhibited significant growth when the medium was supplemented with sucrose and either of the two NDPs (Fig. 2D). Curiosly, this strain also exhibited some growth when the medium was supplemented only with sucrose. This indicates that intracellular provision of NDP is sufficient to allow for activity of this enzyme variant, which confirms model based observations. (Fig. 2D).

## Discussion

Enzyme engineering for the development of industrially suitable biocatalysts entails complex improvements of activity, stability, and other desired features. Even with HTS methods available, the process can become tedious when multiple substitutions are needed to obtain the targeted properties, leading to a combinatorial landscape that can quickly exceed the experimentally possible.

*In vivo* selection systems are generally regarded as simple and low tech methodology to screen large librarys, which size are only limited by transformation efficiency (Neuenschwander et al. 2007; Wu et al. 2022). However, developing effective growth coupled selection systems can be challenging, as the link between enzyme kinetic activity and growth must be carefully established in order to discriminate enhanced variants from the starting enzyme during several rounds of directed evolution. Different tools have been developed to increase selection pressure and allow a wider dynamic range of *in vivo* selection systems, such as the use of regulable promoters, a variety of ribosomal binding sites, and even protein degradation tags (Nearmnala et al. 2021; Neuenschwander et al. 2007; Wu et al. 2022). These tools remain to be further implemented in our selection platform in order to be able to enhance SuSy activity by directed evolution selecting for faster growth rates.

The challenge to use *in vivo* selection systems becomes especially difficult when aiming to improve enzyme stability for its use in *in vitro* biocatalysis, given that reaction conditions (e.g., high substrate load, presence of organic solvent, pH, and temperature) are often impossible to mimic in an intracellular environment. However, the development of computational approaches fed with big data generated from growth coupled platforms, such as global multi mutant analysis (GMMA) (Johansson et al. 2023) opens the door for such endeavours. GMMA relys on the fact that beneficial mutations, even if they do not result in a discernible phenotype, can be identified by their ability to compensate for deleterious mutations, which do have a phenotype (Norrild et al. 2022). By integrating the information of phenotype (in this case, growth/no growth) and genotype (mutations in the gene) of a large library of multiple mutated variants, it would be possible to assign the effects of individual mutations.

SuSys hold potential to become an industrial biocatalysts when coupled with UGTs for the production of valuable glycosides with diverse uses and bioactive properties. However, lack of a robust variant, ideally in the form of high kinetic stability in the prescense of organic solvents and high aglycon loads, prevent its widespread application. Moreover, lack of efficient HTS methods for this enzyme class hinders the engineering efforts towards desired properties, limiting tests to a reduced number of rational designs. Our work presents a solution to overcome these limitations. The proposed procedure for the stability engineering of any desired SuSy using our strain platform and GMMA is summarized in Supplemental Fig. 1.

TDP activated sugars are the most structurally diverse class of nucleotide sugars found in nature, and the preferred option in the biosynthesis of most of the bacterial glycosylated natural products (Thibodeaux et al. 2008). In fact, the *Bacillus licheniformis* (UDP) glycosyltransferase YjiC has already been engineered to increase specificity towards TDP-glucose, as it has been hypothetised that microbial production of glycosides would be favoured given that TDP-sugars are abundantly present in microbial cytosol (Cho et al. 2019). In this context, a SuSy with specificity towards TDP could play an important role for the recycling of sugar donors within microbial cell factories for *in vivo* production of glycosides, as currently proosed in the recombinant production of steviol glycosides (Dalgaard Mikkelsen et al. 2012). The growth coupled selection platform presented in this work is readily applicable for the engineering of SuSy NDP specificity, by providing an alternative NDP in the culture medium.

In summary, our selection design works as a flexible platform that can be used for the engineering of different features of SuSy, and other industrially relevant sucrose utilizing enzymes such as invertases (involved in the production of high fructose syrup), or β-Fructofuranosidase (for fucosyl-oligosaccharides synthesis).

### Electronic supplementary material

Below is the link to the electronic supplementary material.

Supplementary Material 1

## Data Availability

The strain SDT 365 is readily available upon request. All data generated or analyzed during this study are included in this published article (and its Supporting Information files).

## Abbreviations

SuSy: Sucrose synthase
HTS: High throughput screening
UDP: Uridine diphosphate
ADP: Adenosine diphosphate
TDP: Thymidine diphosphate
NDP: Nucleoside diphosphate
UGT: UDP-dependent glycosyltransferase
UDP-Glc: Uridine 5’-diphosphate glucose
DMSO: Dimethyl sulfoxide
GMMA: Global multi mutant analysis
UDP: Uridine diphosphate
E. coli: Escherichia coli
