# Supplementary material for: A growth selection system for sucrose synthases (SuSy): design and test
Source: AMB Express. 2024 Jun 12;14:70. doi: 10.1186/s13568-024-01727-y (PMC11169191; doi:10.1186/s13568-024-01727-y)
Supplement: Supplementary file 1 — Supplementary Material 1 [file 13568_2024_1727_MOESM1_ESM.docx]

***A growth selection system for sucrose synthases (SuSy): design and test.***

Gonzalo N. Bidart^1^, Se Hyeuk^1^, Tobias Benedikt Alter^1,2^, Lei Yang^1^, Ditte Hededam Welner^1,*^

*Author information:*

**Gonzalo N. Bidart** *(*[*gonbid@biosustain.dtu.dk*](mailto:gonbid@biosustain.dtu.dk)*)*

**Se Hyeuk** *(*[*hyki@biosustain.dtu.dk*](mailto:hyki@biosustain.dtu.dk)*)*

**Tobias Benedikt Alter** *(*[*tobias.alter@rwth-aachen.de*](mailto:tobias.alter@rwth-aachen.de)*)*

**Lei Yang** *(*[*leiya@biosustain.dtu.dk*](mailto:leiya@biosustain.dtu.dk)*)*

**Ditte Hededam Welner** *(*[*diwel@biosustain.dtu.dk*](mailto:diwel@biosustain.dtu.dk)*) Phone: +45 93513498*

^1^The Novo Nordisk Center for Biosustainability, Technical University of Denmark, Kemitorvet 220, DK-2800 Kgs. Lyngby, Denmark.

^2^present address: RWTH Aachen University, Templergraben 55, 52062 Aachen, Germany
^*^Corresponding author

**Supplementary information**

[**Supplementary Table 1 – Primers used in this study** 2](#_Toc162999274)

[**Supplementary Figure S1** 3](#_Toc162999275)

[**Supplementary Notebook 1 – Jupyter notebook.** 4](#_Toc162999276)

# **Supplementary Table 1 – Primers used in this study**

| No. | Sequence 5’-3’ | Description |
| --- | --- | --- |
| pMPT7-Fw | AGTAATAAAUCGCACTCACTGCTTCACA | Forward primer to amplify backbone fragment from pMTL61110 |
| pMPT7-Rv | AGAAAACCUCCTTAGCATGATTAAGATGTTTCAG | Reverse primer to amplify backbone fragment from pMTL61110 |
| SuSy Sec Fw | TTGTACCTATAATGTGTGGAGGG | Forward primer for sequencing inserted gene on pMTP7. |
| SuSy Sec Rv | CCCGGCGGCAACCGAGCGTTCTG | Reverse primer for sequencing inserted gene on pMTP7. |
| GmSuSy-Fw | AGGTTTTCUAATGGGCAGCAGCCATCAT | Forward primer to amplify *GmSuSy* |
| GmSuSy-Rv | ATTTATTACUCAGCAGCAAGGGGCACAG | Reverse primer to amplify *GmSuSy* |
| AcSuSy-Fw | AGGTTTTCUAATGGGTAGTAGCCATCAT | Forward primer to amplify *AcSuSy* |
| AcSuSy-Rv | ATTTATTAUTCCATAGGCACGGCATGTG | Reverse primer to amplify *AcSuSy* |
| SD_PR356 | ATTTCTACUCTTGTAGATTTATTTGAATCAGCATTTTGAGGCCGGCATGGTCCCAGCCTCCTCGCTGGCGCCGGCTGGGCAACATGCTTCGGCATGGCGAATGGGACATCCCAGAAAAGACCCGTC | Forward primer to construct pSD85 |
| SD_PR357 | AGTAGAAAUTCCAAAGGTCTTTTGACCTTCCACGGATGATGTATCGAATCTACAAGAGTAGAAATTCCAAAGGTCTTTTGACGACGAGCTTACTCGTTTCGTCCTCACGGACTCATCAGGTCAAAACTAGTATTATACCTAGGACTG | Reverse primer to construct pSD85 |
| SD_PR361 | AGCCAGAGTGCTCCATGTTGCAGCACAGCCACTCCGTGGGAGGCACAATCGAGATTGCGTCATTTTAATCATCCTGGTTAAGCAAATTTG | MAGE oligo to knockout *cscA* |
| SD_PR364 | GTTCCACTTACGGGAGATTAACCGCTCCTACGCAAGCTTTGGAAAGTGCAATCCGGTTAGATACTACGACTAAACGACTCATAGTCATC | MAGE oligo to knockout *otsA* |
| SD_PR359 | CCTAAAACCCATACTTTGGC | *cscA* forward flanking |
| SD_PR428 | TTAACCCAGTAGCCAGAG | *cscA* reverse flanking |
| SD_PR362 | AAGCATCATTTCGATTTGGTG | *otsA* forward flanking |
| SD_PR363 | AACTCAGGCATCATGGCG | *otsA* reverse flanking |

# **Supplementary Figure S1**


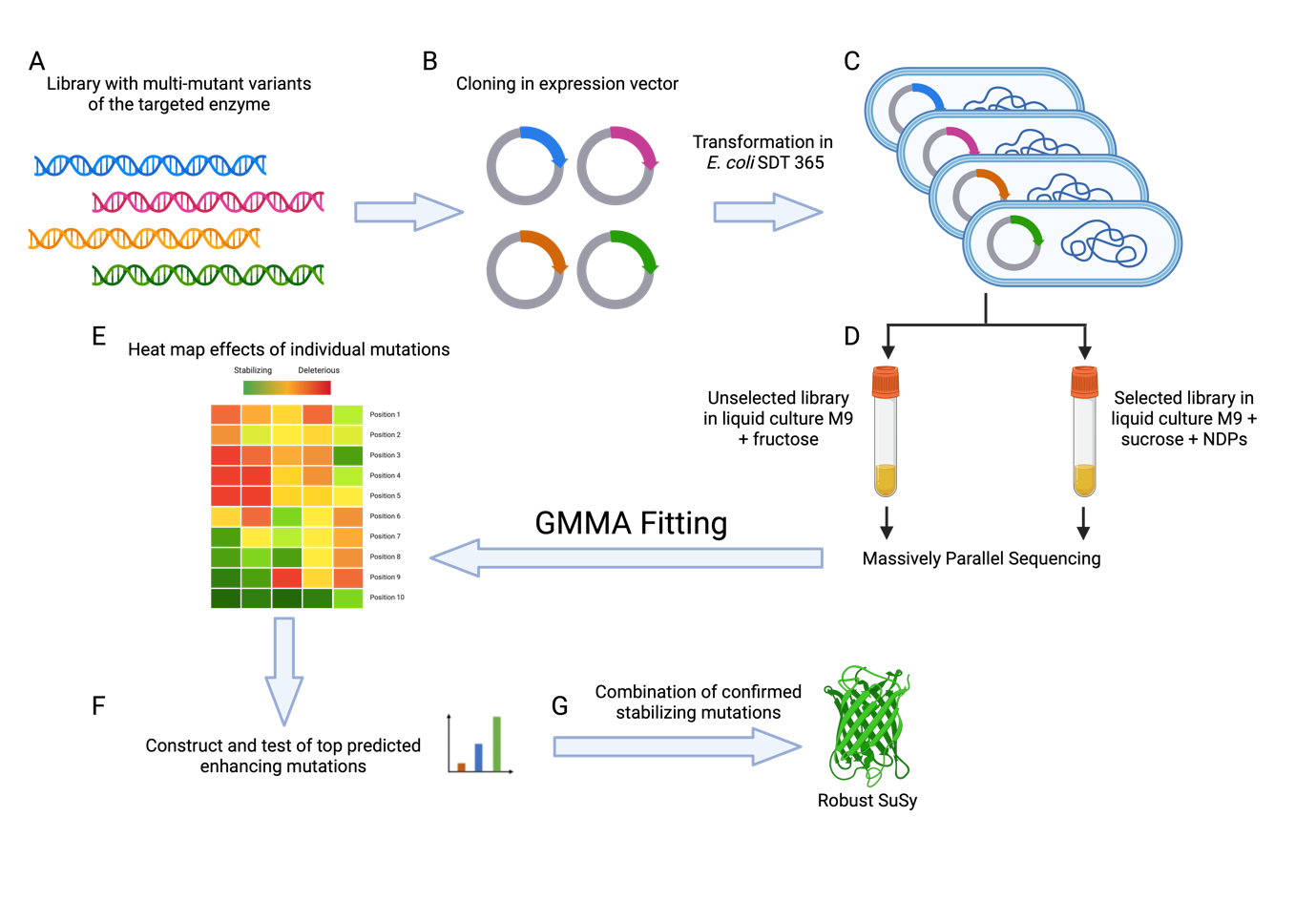


**Supplementary Figure S1.** General procedure for SuSy engineering using the growth-based selection platform and GMMA. A) Design and construct library of multi-mutant variants of the targeted SuSy. B) Clone into appropriate vector. C) SDT365 are transformed with the library. D) Grow the obtained library in unselected and selected conditions, and do massively parallel sequencing of both populations. E) GMMA fitting. F) Characterization of predicted enhancing mutations. G) Combination of tested enhancing mutations

# **Supplementary Notebook 1 – Jupyter notebook.**

A Jupyter notebook is available as a supplementary file containing all applied metabolic model adaptions, relevant simulations, and analyses. The script was tested with Python 3.10 and requires the cobrapy package (version 0.29.0) as well as a valid installation of the Gurobi optimization suite including the Python package gurobipy (version 10.0.1). All Python dependencies and requirements can be retrieved from the environment.yml file.
